# Construction and Validation of Nomograms Predicting Survival in Triple-Negative Breast Cancer Patients of Childbearing Age

**DOI:** 10.3389/fonc.2020.636549

**Published:** 2021-02-08

**Authors:** Xiang Cui, Deba Song, Xiaoxu Li

**Affiliations:** Department of Thyroid and Breast Surgery, The First People’s Hospital of Shangqiu, Shangqiu, China

**Keywords:** nomogram, triple-negative breast cancer, overall survival, breast cancer-specific survival, SEER, prognosis, prediction, childbearing age

## Abstract

**Background:**

Triple-negative breast cancer (TNBC) is one of the most aggressive subtypes of breast cancer with poorest clinical outcomes. Patients of childbearing age have a higher probability of TNBC diagnosis, with more demands on maintenance and restoration of physical and psychosocial function. This study aimed to design effective and comprehensive nomograms to predict survival in these patients.

**Methods:**

We used the SEER database to identify patients with TNBC aged between 18 and 45 and randomly classified these patients into a training (n=2,296) and a validation (n=2,297) cohort. Nomograms for estimating overall survival (OS) and breast cancer-specific survival (BCSS) were generated based on multivariate Cox proportional hazards models and competing-risk models in the training cohort. The performances of the nomograms were quantified in the validation cohort using calibration curves, time-dependent receiver operating characteristic (ROC) curves and Harrell’s concordance index (C-index).

**Results:**

A total of 4,593 TNBC patients of childbearing age were enrolled. Four prognostic factors for OS and six for BCSS were identified and incorporated to construct nomograms. In the validation cohort, calibration curves showed excellent agreement between nomogram-predicted and actual survival data. The nomograms also achieved relatively high Harrell’s C-indexes and areas under the time-dependent ROC curves for estimating OS and BCSS in both training and validation cohorts.

**Conclusions:**

Independent prognostic factors were identified, and used to develop nomograms to predict OS and BCSS in childbearing-age patients with TNBC. These models could enable individualized risk estimation and risk-adapted treatment for these patients.

## Introduction

Breast cancer is the most prevalent malignancy among females, which ranks first in new cases and second in deaths according to estimation from the American Cancer Society in 2019 (1). And as of 2019, there were more than 3.8 million women with a history of invasive breast cancer in the United States (2). Triple-negative breast cancer (TNBC) is a subset of breast cancer that lacks expression of estrogen receptor (ER), progesterone receptor (PR), and human epidermal growth factor receptor 2 (HER2). TNBC represents one of the most aggressive subtypes of breast cancer and remains the most challenging subtype to treat (3). The proportion of patients of childbearing age was higher in TNBC than in other breast cancer subtypes (4), and childbearing females were also more likely to be diagnosed with TNBC than with the other subtypes (5). In addition, childbearing-age patients have relatively different demographics and clinicopathologic characteristics and treatment strategies compared with patients in other physiological stages (6–8). Childbearing age refers to nearly 30 years after a woman reaches the age of 18. In this period, the reproductive and endocrine functions of the ovaries attained full growth or maturity, and the mammary glands undergo periodic changes under the regulation of ovarian hormones. The ovarian dysfunction, delayed childbearing, inability to breastfeed, and job changes, which may result from cancer treatment, have a tremendous impact on the physical, psychosocial well-being of these patients, resulting a reduction of disability adjusted life years (DALYs) (9–12). Therefore, it is of great significance to differentiate these patients with different risk of death, especially breast cancer specific death, and implement different treatment strategies.

With the increased use of neoadjuvant chemotherapy in TNBC patients, pathologic response has been recognized as an important prognostic factor (13). However, neoadjuvant has adverse therapeutic effects and takes time, which have an impact on the childbearing patients’ willingness to treatment (14). Even worse, some non-pathologic responding patients do not benefit from it and may delay prompt treatment. So, if the level of risk can be identified based on other characteristics, it will facilitate the identification and implementation of a tailored treatment. Our previous study described the molecular characteristics of TNBC patients of childbearing age, which provide a rationale for clinical management (15). However, in clinical practice, clinicopathologic characteristics are more accessible for clinicians than molecular profiling. Thus, the urgent clinical need for risk estimation prompted us to construct a clinicopathological information-based model for predicting survival in childbearing-age TNBC patients.

Nomograms are reliable and effective tools to quantify individual risk by incorporating and illustrating multiple important prognostic factors. They performed well in predicting survival in a variety of cancer types (16). In addition to nomogram for all four subtypes of breast cancer (17), researchers established specific nomograms for different histological subtypes (18–23), clinical subtypes (24–26), metastatic status (27–30), and age group (31–35) of breast cancer. However, to the best of our knowledge, nomograms for predicting the survival of childbearing-age patients with TNBC have not been reported. In this study, we aimed to formulate comprehensive nomograms based on complete clinical data selected from the Surveillance, Epidemiology, and End Results (SEER) database to estimate survival in TNBC patients of childbearing age.

## Materials and Methods

### Data Source and Patient Screening

Data were extracted from the SEER*Stat version 8.3.6.1, SEER 18 Cancer Registry [1976-2016] (with additional treatment fields) of the National Cancer Institute. The following criteria were used to identify eligible patients: female gender; age of 18–45 years at diagnosis; diagnosed between 2010 and 2015; pathologically confirmed invasive ductal carcinoma of the breast (ICD-O-3 8500/3); diagnosis confirmed by positive histology and not by autopsy or a death certificate; breast cancer as the first and only primary tumor; unilateral breast cancer; adjusted AJCC stage I-III; histological grade I-III; known tumor size; known regional lymph nodes status; and ER, PR, and HER2 negativity. Since the HER2 status was not recorded in the database until 2010, patients diagnosed before 2010 were not included. Patients diagnosed after 2015 were also excluded to ensure adequate follow-up time. Exclusion criteria were as follows: no record of regional lymph node status or tumor size; Paget’s disease and inflammatory breast cancer; incomplete survival data and unspecified tumor laterality or location information; survival month less than 1. Eventually, 4,593 patients were included after the screening. The following data were collected and transformed into categorical variables: age, race, marital status, laterality, grade, location, tumor size, positive lymph nodes, breast surgery, radiation, and chemotherapy.

### Identification of Prognostic Factors in the Training Cohort

Patients were randomly classified into a training and a validation cohort at a 1:1 ratio (36). The primary endpoint were overall survival (OS) and breast cancer-specific survival (BCSS). OS was defined as the interval from breast cancer diagnosis to the last follow-up or death from any cause. BCSS was define as the interval from the time of diagnosis to last follow-up or death from breast cancer. Independent prognostic factors for OS were identified by multivariate Cox proportional hazards models, and the results were reported using hazard ratios (HRs) and 95% confidence intervals (CIs). The cumulative incidence rates of breast cancer specific death (CIBCSD) were calculated based on competing-risk models, and differences among groups were assessed by the Gray’s test (37, 38). In the competing-risk regression model, deaths from non-breast cancer specific causes were considered as competing risks.

### Model Construction in the Training Cohort

Two nomograms were constructed to predict survival in the training cohort (39). Independent prognostic factors in multivariate Cox proportional hazards models were used to construct the nomogram for 3- and 5-year OS. Factors associated with CIBCSD in the competing-risk models were used to build the BCSS nomogram. The BCSS nomogram was also constructed based on the Cox regression model, in which patients succumbing to non-breast cancer specific causes were considered to be censored.

### Model Validation in Both Cohorts

The nomograms were validated in both training and validation cohorts. First, the predictive accuracies of the nomograms were validated by bootstrapping with 1000 repetitions, and the discriminative ability was quantified by the concordance index (C-index). The C-index ranges from 0.5 (occurring by random chance) to 1.0 (perfectly correct discrimination). Second, calibration curves were generated to obtain nomogram-predicted survival, which is then compared with the corresponding Kaplan-Meier estimates. Third, according to the nomogram, we calculated the total points for all patients (40). The predictive precision of the risk score as a continuous variable was evaluated by time-dependent receiver operating characteristic (ROC) curves, and areas under the curves (AUCs) were used as the criterion (41). The ROC curves plotted the predictive sensitivity and specificity; a larger AUC (range 0.5~1.0) reflected a more accurate prediction. Finally, to demonstrate the clinical values of the nomograms that included all meaningful variables, two normal TNBC patients were assessed as examples.

### Statistical Analysis

All statistical analyses were performed with STATA (version 14.1) and R (version 3.6.1). The R packages including caret, rms, cmprsk, survivalROC, and nomogramFormula were used. Statistical significance was defined as P < 0.05.

## Results

### Patient Characteristics

A total of 4,593 patients from the SEER program were enrolled in our study. The demographics and clinicopathologic characteristics of these patients are listed in **Table 1**. Among these patients, median follow-up months were 37 months (25%–75%, 22–58 months). Nearly half of them were aged between 40 and 45 (45.8%), while those between 35 and 40 (29.0%), and less than 35 (25.2%) composed the remaining half. Most of the patients were white (69.4%) and more than half of the patients were unmarried (58.8%). All assessed factors showed similar distribution between the training and validation cohorts.

**Table 1:** Patients’ demographics and clinicopathologic characteristics.

	All patients	Training cohort	Validation cohort
	N=4,593 (%)	N=2,296 (%)	N=2,297 (%)
**Median follow-up months (IQR)**	37 (22–58)		
**Age (years)**			
≤ 35	1158 (25.2)	571 (24.9)	587 (25.6)
35–40	1331 (29.0)	670 (29.2)	661 (28.8)
40–45	2104 (45.8)	1055 (45.9)	1049 (45.7)
**Race**			
White	3188 (69.4)	1571 (68.4)	1617 (70.4)
Black	915 (19.9)	471 (20.5)	444 (19.3)
Others^#^	490 (10.7)	254 (11.1)	236 (10.3)
**Marital status^$^**			
Married	1892 (41.2)	952 (41.5)	940 (40.9)
Unmarried	2701 (58.8)	1344 (58.5)	1357 (59.1)
**Laterality**			
Left	2355 (51.3)	1179 (51.4)	1176 (51.2)
Right	2238 (48.7)	1117 (48.6)	1121 (48.8)
**Grade**			
I	24 (0.5)	14 (0.6)	10 (0.4)
II	426 (9.3)	211 (9.2)	215 (9.4)
III	4143 (90.2)	2071 (90.2)	2072 (90.2)
Location*			
Central	95 (2.1)	50 (2.2)	45 (2.0)
Inner	910 (19.8)	439 (19.1)	471 (20.5)
Outer	2167 (47.2)	1111 (48.4)	1056 (46.0)
Tail	41 (0.9)	21 (0.9)	20 (0.9)
Overlap	1380 (30.0)	675 (29.4)	705 (30.7)
**Tumor Size (cm)**			
≤ 2	1510 (32.9)	746 (32.5)	764 (33.3)
2–5	2455 (53.5)	1222 (53.2)	1233 (53.7)
> 5	628 (13.7)	328 (14.3)	300 (13.1)
**Positive lymph nodes**			
0	2752 (59.9)	1370 (59.7)	1382 (60.2)
1–3	1017 (22.1)	526 (22.9)	491 (21.4)
> 3	824 (17.9)	400 (17.4)	424 (18.5)
**Breast Surgery**			
BCS	1924 (41.9)	934 (40.7)	990 (43.1)
Mastectomy	2669 (58.1)	1362 (59.3)	1307 (56.9)
**Radiation**			
Yes	2144 (46.7)	1062 (46.3)	1082 (47.1)
No	2449 (53.3)	1234 (53.7)	1215 (52.9)
**Chemotherapy**			
Yes	4183 (91.1)	2091 (91.1)	2092 (91.1)
No/Unknown	410 (8.9)	205 (8.9)	205 (8.9)

^#^American Indian/AK Native, Asian/Pacific Islander.

^$^Unmarried included single (never married), widowed, separated, divorced, and unmarried or domestic partner.

*Central, codes C50.0 and C50.1; Inner, codes C50.2 and C50.3; Outer, codes C50.4 and C50.5; Tail, code C50.6; Overlap, codes C50.8 and C50.9. From SEER Coding Guidelines Breast 2018 manual, coding guideline breast C500–C509.

BCS, breast conservation surgery; IQR, interquartile range.

### Factors Associated With Overall Survival in the Training Cohort

In univariate Cox analysis, race, marital status, tumor location, tumor size, number of positive lymph nodes and breast surgery type were significantly correlated with OS (all P < 0.001 except for race and breast surgery, with P = 0.009 and P = 0.003, respectively). These prognostic factors were included in multivariate Cox analysis. The results confirmed that unmarried status, overlapped tumor location, large tumor, and more positive lymph nodes were independent adverse prognostic factors (**Table 2**). These variables were included in a weighted scoring system to estimate 3- and 5-year OS.

**Table 2:** Univariate and multivariate analyses of overall survival in the training cohort.

	Training cohort		
	Univariate	Multivariate	P value
	P value	HR (95% CI)	
**Age (years)**	0.762		
≤ 35			
35–40			
40–45			
**Race**	**0.009**		
White		Ref.	**-**
Black		1.06 (0.83–1.37)	0.621
Others^#^		0.73 (0.50–1.08)	0.114
**Marital status^$^**	**<0.001**		
Married		Ref.	**-**
Unmarried		1.49 (1.19–1.85)	**<0.001**
**Laterality**	0.637		
Left			
Right			
**Grade**	0.134		
I			
II			
III			
Location*	**<0.001**		
Central		1.51 (0.90–2.55)	0.190
Inner		0.92 (0.67–1.26)	0.594
Outer		Ref.	**-**
Tail		0.62 (0.15–2.55)	0.503
Overlap		1.22 (1.03–1.81)	**0.045**
**Tumor Size (cm)**	**<0.001**		
≤ 2		0.62 (0.46–0.82)	**0.001**
2–5		Ref.	–
>5		2.02 (1.59–2.57)	**<0.001**
**Positive lymph nodes**	**<0.001**		
0		Ref.	–
1–3		2.54 (1.94–3.32)	**<0.001**
> 3		4.47 (3.44–5.82)	**<0.001**
**Breast Surgery**	**0.003**		
BCS		Ref.	–
Mastectomy		1.20 (0.96–1.51)	0.111
**Radiation**	0.169		
Yes			
No			
**Chemotherapy**	0.782		
Yes			
No/Unknown			

^#^American Indian/AK Native, Asian/Pacific Islander.

^$^Unmarried included single (never married), widowed, separated, divorced, and unmarried or domestic partner.

*Central, codes C50.0 and C50.1; Inner, codes C50.2 and C50.3; Outer, codes C50.4 and C50.5; Tail, code C50.6; Overlap, code C50.8. From SEER Coding Guidelines Breast 2018 manual, coding guideline breast C500–C509.

BCS, breast conservation surgery; CI, confidence interval; HR, hazard ratio; Ref., Reference.

### Factors Associated With Breast Cancer-Specific Survival in the Training Cohort

To identify prognostic factors associated with BCSS, we determined CIBCSD and cumulative incidence of non-breast cancer specific death (CINBCSD) based on the developed competing-risk models. At 3 and 5 years after diagnosis, CIBCSD rates in the training cohort were 0.144 and 0.195, respectively, while CINBCSD rates were 0.011 and 0.020, respectively. Estimates of CIBCSD and other causes according to the clinicopathological variables are shown in **Table 3**. Black patients had the highest CIBCSD (0.192 and 0.246 for 3 and 5 years, respectively), while white and patients of other race had lower CIBCSD (white, 0.133 and 0.187 for 3 and 5 years, respectively; other race, 0.122 and 0.142 for 3 and 5 years, respectively; P = 0.006). Other factors significantly associated with CIBCSD are marital status, tumor size, tumor location, lymph node status, and surgery type (all P < 0.001). All these factors were used to construct a nomogram to predict 3- and 5-year BCSS.

**Table 3:** Three- and 5-year cumulative incidence rates of death among patients in the training cohort.

	Cumulative incidence of breast cancer-specific death			Cumulative incidence of non-breast cancer-specific death		
	3-year	5-year	P value	3-year	5-year	P value
**Age (years)**			0.586			0.627
≤ 35	0.147	0.173		0.013	0.016	
35–40	0.162	0.216		0.009	0.016	
40–45	0.131	0.192		0.010	0.024	
**Race**			**0.006**			0.383
White	0.133	0.187		0.001	0.023	
Black	0.192	0.246		0.011	0.015	
Others^#^	0.122	0.142		0.004	0.004	
**Marital status^$^**			**<0.001**			0.210
Married	0.113	0.153		0.009	0.017	
Unmarried	0.188	0.253		0.014	0.023	
**Laterality**			0.688			0.813
Left	0.137	0.200		0.014	0.019	
Right	0.152	0.190		0.007	0.021	
**Grade**			0.368			0.643
I	0.000	0.000		0.000	0.000	
II	0.123	0.190		0.014	0.034	
III	0.147	0.197		0.010	0.018	
Location*			**<0.001**			0.958
Central	0.293	0.378		0.021	0.021	
Inner	0.099	0.148		0.005	0.019	
Outer	0.134	0.187		0.011	0.022	
Tail	0.056	0.134		0.000	0.000	
Overlap	0.182	0.227		0.012	0.015	
**Tumor Size (cm)**			**<0.001**			0.665
≤ 2	0.065	0.093		0.009	0.017	
2–5	0.133	0.191		0.011	0.020	
>5	0.356	0.432		0.015	0.024	
**Positive lymph nodes**			**<0.001**			0.356
0	0.059	0.099		0.006	0.019	
1–3	0.203	0.265		0.013	0.016	
> 3	0.359	0.428		0.022	0.026	
**Breast Surgery**			**<0.001**			0.474
BCS	0.111	0.146		0.010	0.015	
Mastectomy	0.166	0.227		0.011	0.022	
**Radiation**			0.088		0.395	0.395
Yes	0.166	0.216		0.009	0.016	
No	0.125	0.176		0.012	0.023	
**Chemotherapy**			0.444			**0.001**
Yes	0.146	0.199		0.009	0.015	
No/Unknown	0.125	0.162		0.024	0.058	
All Patients	0.144	0.195		0.011	0.020	

^#^American Indian/AK Native, Asian/Pacific Islander.

^$^Unmarried included single (never married), widowed, separated, divorced, and unmarried or domestic partner.

*Central, codes C50.0 and C50.1; Inner, codes C50.2 and C50.3; Outer, codes C50.4 and C50.5; Tail, code C50.6; Overlap, code C50.8. From SEER Coding Guidelines Breast 2018 manual, coding guideline breast C500–C509.

BCS, breast conservation surgery.

### Nomograms

**Figure 1:**
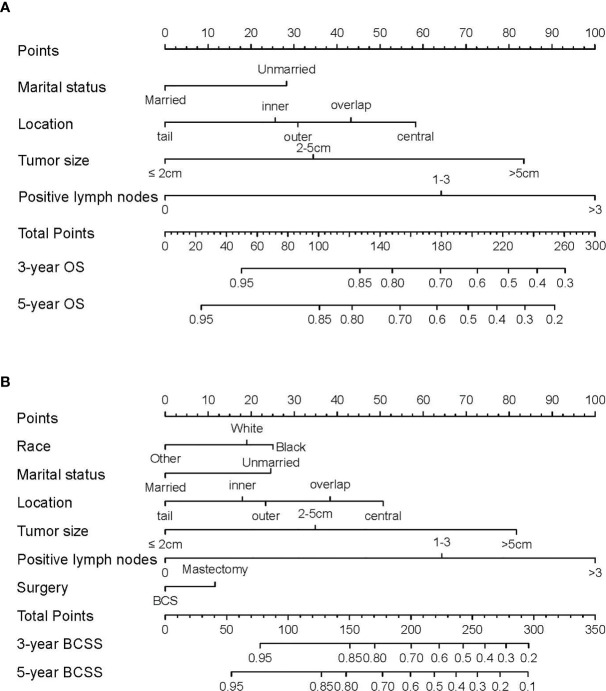
Nomograms predicting 3- and 5-year **(A)** overall survival (OS) and **(B)** breast cancer-specific survival (BCSS) in TNBC patients of childbearing age. Instructions for nomogram use were as follows. First, assign points to each characteristic for a given patient by drawing a vertical line from that variable to the points scale. Then, sum all the points and draw a vertical line from the total points scale to the 3- and 5-year OS or BCSS to obtain the likelihood of 3- or 5-year survival.

Based on the prognostic factors identified in the training cohort, nomograms were formulated to predict 3- and 5-year OS and BCSS (**Figure 1**). To clarify the applications of these nomograms, two representative TNBC patients were assessed. Both patients were married, underwent surgery, and diagnosed with grade III, invasive ductal carcinoma with outer location. The first patient was a 36-year-old white patient diagnosed with a tumor of 1.5 cm in diameter and without positive lymph node, while the second was a 37-year-old patient of other race (American Indian/AK Native, Asian/Pacific Islander) diagnosed with a tumor of 5.5 cm in diameter and with 5 positive lymph nodes. According to the nomograms, the first patient scored 59.1 and 47.9 in the OS and BCSS nomograms, respectively, which indicated that her odds of 3- and 5-year OS were greater than 0.85, and those of 3- and 5-year BCSS were greater than 0.95. Scores of the second patient in OS and BCSS nomograms were 214.3 and 205.1, respectively, which indicated that the odds of both 3- and 5-year OS and BCSS were less than 0.7. These results can help to guide individualized treatment for these two patients.

### Model Validation

**Figure 2:**
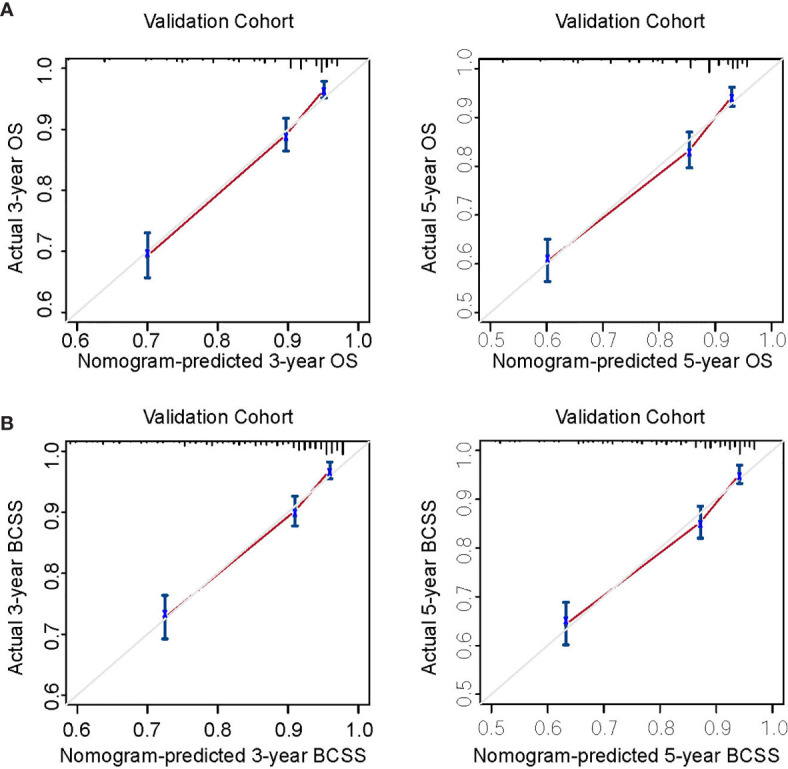
Calibration curves for external validation. **(A)** Nomogram calibration curves for 3- and 5-year overall survival (OS). **(B)** Nomogram calibration curves for 3- and 5-year breast cancer-specific survival (BCSS). X-axis, nomogram-predicted survival; Y-axis, actual survival.

**Figure 3:**
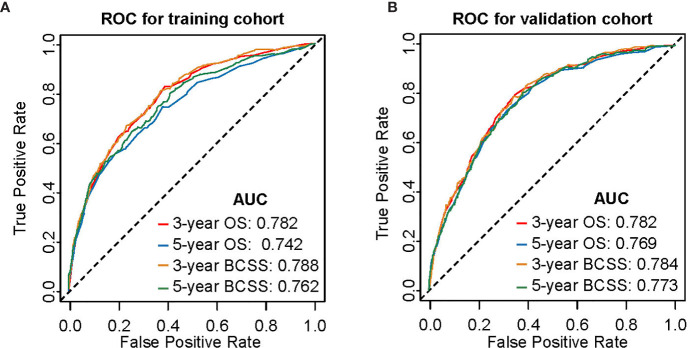
Time-dependent receiver operating characteristic (ROC) curves for predicting 3- and 5-year OS and BCSS. **(A)** Internal validation in the training cohort. **(B)** External validation in the validation cohort. AUC, area under curve; OS, overall survival; BCSS, breast cancer-specific survival.

Nomogram validation was processed in both the training and validation cohorts. In the training cohort, the Harrell’s C-indexes for the nomograms for predicting OS and BCSS were 0.766 and 0.776, respectively. In the validation cohort, the C-indexes were slightly lower, i.e., 0.763 and 0.765, respectively (**Table S1**). The external and internal calibration curves were shown in **Figure 2** and **Figure S1**, which demonstrated an excellent agreement between the actual and nomogram-predicted survival rates. The time-dependent ROC curves for predicting 3- and 5-year OS and BCSS in the training and validation cohorts were presented in **Figure 3**. With the risk score as a continuous variable, the AUCs for 3- and 5-year OS and BCSS predictions were all above 0.74. These results demonstrated that the nomograms were useful tools for the prediction of survival in TNBC patients of childbearing age.

## Discussion

In the light of the relatively high mobility and invasiveness of tumor, strong desire to preserve and restore physiological and social functions, high requirements for quality of life, and unique clinicopathological features of TNBC patients of childbearing age, a brief nomogram based on follow-up data of a large cohort for predicting OS and BCSS should be quite practical in clinic. Although nomograms predicting survival of patients with TNBC or of a specific age group have been reported (20, 22, 31–33, 35), there is no nomogram for TNBC patients of childbearing age, a period of highly active physiological and social function. Using data from the SEER program, we established reliable nomograms to predict the 3- and 5-year OS and BCSS of these patients based on Cox regression and competing-risk models. The calibration curves, time-dependent ROC analysis and Harrell’s C-indexes demonstrated satisfactory performances of our nomograms. Therefore, our nomograms can be used for personalized risk prediction and to guide treatment for TNBC patients of childbearing age.

In the current study, several demographics and clinicopathologic characteristics were shown to be prognosis factors of 3- and 5-year OS and BCSS, including marital status, tumor location, tumor size, number of positive lymph nodes, race and surgery, corroborating previous studies of TNBC patients (42–46). The primary tumor site is considered as an important independent prognostic factor of breast cancer, and several studies have shown that tumor location in lower inner zone suggests a poor prognosis (47, 48). In the univariate analysis of OS in the training cohort, OS was significantly different among the five groups of tumor location. In multivariate analysis, though only the “overlap” site was significantly associated with poor prognosis, the hazard ratios of various groups confirmed the impact of tumor location on OS. Therefore, this factor was incorporated in the nomogram for predicting OS. As for BCSS, different groups of location were significantly different in CIBCSD and showed no differences in CINBCSD, indicating that tumor location is a significant prognosis factor of breast cancer-specific death. According to the CIBCSD of each group, we developed a scoring system to qualify the risk caused by tumor location. Previous studies have shown higher incidence of TNBC among black women compared with other races, which is determined by biological differences and socioeconomic factors (45, 49). In addition, African ethnicity is a significant and independent predictor of poor outcome (50). In this study, race was a significant but not independent prognosis factor of OS. However, regarding BCSS, black women had higher CIBCSD compared with white and “other” patients. Therefore, race was included in our scoring system for BCSS prediction but not for OS prediction.

Despite the above strengths, there were some limitations in this study. First, the undetailed data of adjuvant chemotherapy and radiotherapy in the SEER database cannot distinguish “no treatment” and “unknown if patients received treatment”, which were combined into a group in our study. Moreover, the lack of information on neoadjuvant therapy in SEER database made it impossible to evaluate the relationship between neoadjuvant therapy and survival in this paper. Some other proven prognostic factors of breast cancer in childbearing age, including breastfeeding, adiposity, and oral contraceptive use (51), were also not available in the SEER database. Although more detailed treatment information is available in other databases, i.e., the National Cancer Database (NCDB) database, we chose SEER as our data source because NCDB is a hospital-based rather than population-based database without available BCSS data (52–54). Second, breast cancer that occurs in patients before the age of 45 has a higher potential to result from hereditary causes. Patients with hereditary breast cancer have a higher risk of recurrence and death. However, because of the lack of genetic data in SEER datasets, we cannot incorporate this important factor into our nomograms, which may lead to predictive bias. Third, patients with incomplete clinical information or survival data were excluded from this study, which could result in selection bias. Further, limited by its single data source and retrospective nature, the nomograms should be further validated in other cohorts, and a prospective research should be performed before its clinical application.

## Conclusion

We developed nomograms to predict OS and BCSS in TNBC patients of childbearing age based on a relatively large cohort with detailed follow-up. The nomograms had excellent performances in both training and validation cohorts. It may serve as an efficient tool for clinicians to predict the prognosis of these patients and to guide individualized treatment.

## Data Availability Statement

The original contributions presented in the study are included in the article/**Supplementary Materials**; further inquiries can be directed to the corresponding author.

## Ethics Statement

Since the data were from the SEER database, informed patient consent and ethical approval were not required.

## Author Contributions

XC conceptualized the work, collected, analyzed, and interpreted the date, drafted the article, critically revised the article, and gave the final approval of the version to be published. DS and XL conceptualized the work, critically revised the article, and gave the final approval of the version to be published. All authors contributed to the article and approved the submitted version.

## Funding

This work was supported by grants from the Training Plan of Excellent Talents of The First People’s Hospital of Shangqiu (SQFPH2019). The funder had no role in the study design, data collection and analysis, decision to publish, or preparation of the manuscript.

## Conflict of Interest

The authors declare that the research was conducted in the absence of any commercial or financial relationships that could be construed as a potential conflict of interest.
