# Supplementary material for: Construction and Validation of Nomograms Predicting Survival in Triple-Negative Breast Cancer Patients of Childbearing Age
Source: Front Oncol. 2021 Feb 8;10:636549. doi: 10.3389/fonc.2020.636549 (PMC7898905; doi:10.3389/fonc.2020.636549)
Supplement: Supplementary file 1 [file DataSheet_1.docx]

**Construction and validation of nomograms predicting survival in** **triple-negative breast cancer patients of childbearing age**

Xiang Cui, Deba Song and Xiaoxu Li

Department of Thyroid and Breast Surgery, The First People's Hospital of Shangqiu, Shangqiu, Henan, China.

Correspondence: Xiang Cui, [xiangcui223@163.com](mailto:xiangcui223@163.com)

**SUPPLEMENTARY FIGURE LEGENDS AND TABLES**

**FIGURE S1 |** Internal calibration curves. (A) Nomogram calibration curves for 3-year and 5-year overall survival (OS). (B) Nomogram calibration curves for 3-year and 5-year breast cancer-specific survival (BCSS). X-axis, nomogram-predicted survival; Y-axis, actual survival.

**Table S1** Harrell’s C-indexes for the nomograms in predicting OS and BCSS

|  | **Training cohort** | | **Validation cohort** | |
| --- | --- | --- | --- | --- |
|  | OS | BCSS | OS | BCSS |
| **Harrell’s C-index** | 0.766 | 0.776 | 0.763 | 0.765 |
