# Supplementary figures and images for: Construction and Validation of Nomograms Predicting Survival in Triple-Negative Breast Cancer Patients of Childbearing Age
Source: Front Oncol. 2021 Feb 8;10:636549. doi: 10.3389/fonc.2020.636549 (PMC7898905; doi:10.3389/fonc.2020.636549)

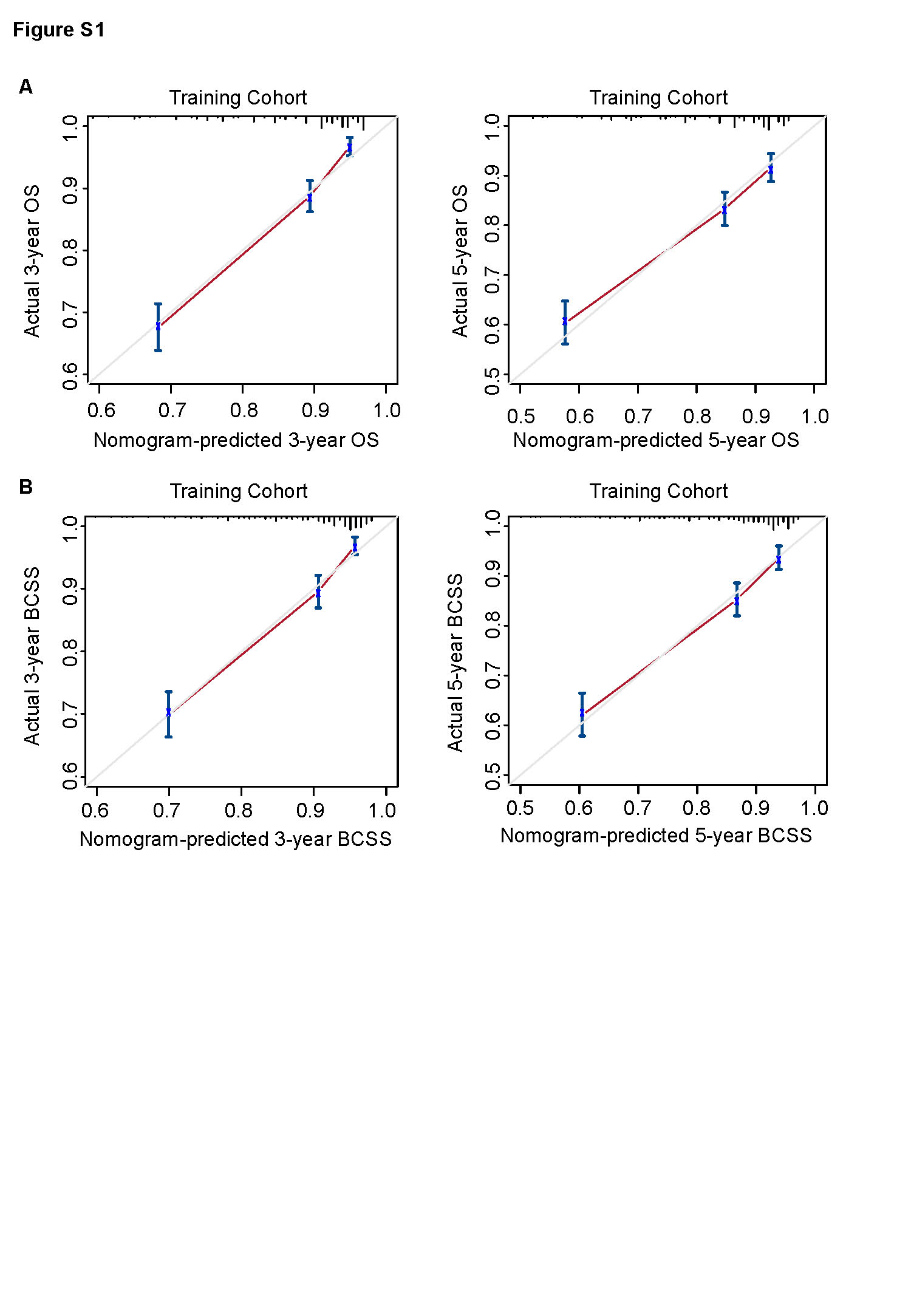

Supplement: Supplementary file 2 [file Image_1.jpeg]
